# Conversion of graded phosphorylation into switch-like nuclear translocation via autoregulatory mechanisms in ERK signalling

**DOI:** 10.1038/ncomms10485

**Published:** 2016-01-20

**Authors:** Yuki Shindo, Kazunari Iwamoto, Kazunari Mouri, Kayo Hibino, Masaru Tomita, Hidetaka Kosako, Yasushi Sako, Koichi Takahashi

**Affiliations:** 1 Graduate School of Frontier Biosciences, Osaka University, Suita, Osaka 565-0871, Japan; 2 Laboratory for Biochemical Simulation, RIKEN Quantitative Biology Center, Suita, Osaka 565-0874, Japan; 3 Cellular Informatics Laboratory, RIKEN, Wako, Saitama 351-0198, Japan; 4 Laboratory for Cell Signaling Dynamics, RIKEN Quantitative Biology Center, Suita, Osaka 565-0874, Japan; 5 Institute for Advanced Biosciences, Keio University, Tsuruoka, Yamagata 997-0017, Japan; 6 Division of Cell Signaling, Fujii Memorial Institute of Medical Sciences, Tokushima University, Tokushima 770-8503, Japan

## Abstract

The phosphorylation cascade in the extracellular signal-regulated kinase (ERK) pathway is a versatile reaction network motif that can potentially act as a switch, oscillator or memory. Nevertheless, there is accumulating evidence that the phosphorylation response is mostly linear to extracellular signals in mammalian cells. Here we find that subsequent nuclear translocation gives rise to a switch-like increase in nuclear ERK concentration in response to signal input. The switch-like response disappears in the presence of ERK inhibitor, suggesting the existence of autoregulatory mechanisms for ERK nuclear translocation involved in conversion from a graded to a switch-like response. *In vitro* reconstruction of ERK nuclear translocation indicates that ERK-mediated phosphorylation of nucleoporins regulates ERK translocation. A mathematical model and knockdown experiments suggest a contribution of nucleoporins to regulation of the ERK nuclear translocation response. Taken together, this study provides evidence that nuclear translocation with autoregulatory mechanisms acts as a switch in ERK signalling.

## Abstract (web summary)

While ERK signalling can produce switch-like cell behaviour, phosphorylation of ERK increases linearly with extracellular signals. Here, the authors solve this seeming contradiction by showing that nuclear translocation of ERK behaves in a switch-like manner and is controlled by ERK activity.

## Graphical abstract

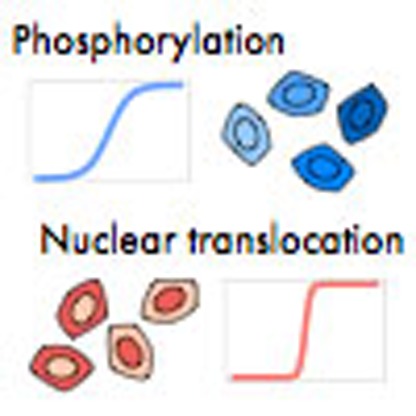

The ERK MAP (mitogen-activated protein) kinase pathway is a master regulator of cell fate decision in eukaryotes^1^,^2^. On receipt of a mitogenic signal, ERK is activated by dual-phosphorylation and catalyses the phosphorylation of numerous proteins, resulting in changes in cell physiology. The ERK pathway consists of a three-tier phosphorylation cascade with multistep reactions and feedback loops, that inherently generate various behaviours including ultrasensitivity, oscillation and memory^3^,^4^,^5^,^6^. An ultrasensitive switch-like response of ERK phosphorylation was actually reported in *Xenopus* oocytes^7^,^8^. Such non-linear properties seem to be appropriate for mediating cellular processes where the state transition emerges.

In contrast, a graded response of ERK phosphorylation was observed in mammalian cells^9^,^10^,^11^,^12^, which suggests that there may be additional mechanisms other than phosphorylation that digitise the graded ERK signal^13^. Although the kinase activity of ERK itself is regulated by dual-phosphorylation on a TEY activation loop, ERK-driven physiological events require more than phosphorylation. Indeed, ERK accumulates in the nucleus after stimulus-induced phosphorylation, and this nuclear translocation is essential for ERK-mediated processes, such as entry into S-phase^14^. Moreover, inhibition of ERK nuclear translocation was recently proposed as a target for anti-cancer therapy^15^. That is, the output of ERK signalling could be understood in terms of the level of nuclear translocation. Recent studies have demonstrated that there is not a simple correlation between the kinetics of phosphorylation and nuclear translocation^16^,^17^, suggesting that regulation of ERK translocation is complex and somewhat distinct from phosphorylation.

Translocation of molecules across the nuclear envelope is mediated by the nuclear pore complex (NPC), which is a large protein complex consisting of ∼30 types of nucleoporins (Nups)^18^. Approximately one third of all Nups contain phenylalanine–glycine repeat regions (FG Nups), which are natively unfolded and form a meshwork or brushwork in the central tube of the NPC that acts as a permeability barrier for non-specific translocation of molecules across the nuclear envelope^19^,^20^. Karyopherins, such as importins and exportins, bind FG Nups and therefore pass through the barrier of the NPC. Indeed, ERK can bind directly to the FG repeat region^21^ and pass through the NPC without carriers^22^,^23^, although a carrier-dependent pathway has also been reported^24^,^25^. Interestingly, several groups reported that Nups are phosphorylated by ERK *in vivo*^26^,^27^,^28^. Furthermore, it has been reported that ERK-mediated phosphorylation of FG Nups affects the nuclear translocation of importin-β (ref. 27). These observations indicate a role of ERK in regulation of nucleocytoplasmic translocation^27^,^29^, which may produce feedback regulation to ERK translocation, although the details remain unclear.

Thus, accumulating evidence suggests the complex nature of ERK nuclear translocation. Here we report that the nuclear translocation behaves like a switch in ERK signalling.

## Results

### Graded response of ERK phosphorylation

**Figure 1:**
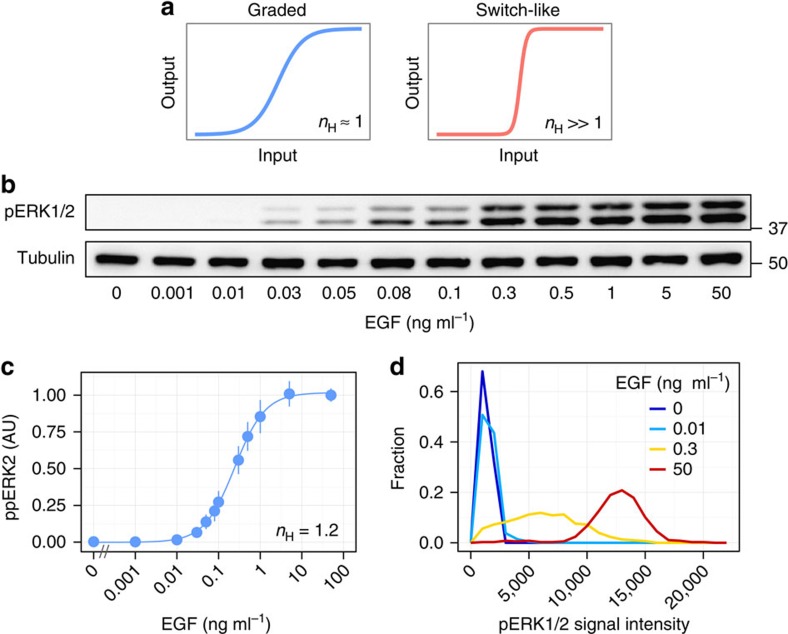
Graded response of EGF-induced ERK phosphorylation in PC12 cells. (**a**) Schematic representation of the graded and switch-like responses of the system. (**b**) Serum-starved cells were stimulated with the indicated concentrations of EGF for 5 min, and the lysates were subjected to western blotting analysis with anti-phospho ERK1/2. (**c**) The means of phosphorylated ERK2 intensity (a.u.) were plotted as a function of EGF concentration with s.e. of three independent experiments. The Hill coefficient was obtained by curve fitting with a Hill function. (**d**) Serum-starved cells were stimulated with the indicated concentrations of EGF for 5 min and then immunostained using anti-phospho ERK1/2 antibody. At least 100 cells were analysed in each condition and histograms of fluorescence signals are shown.

This study was performed to determine the response characteristics of ERK nuclear translocation and the underlying mechanisms that specify the shape of the response (Fig. 1a)^30^. Epidermal growth factor (EGF) stimulation induces transient ERK phosphorylation and nuclear translocation in PC12 cells^31^. We first determined the dose–response relation in terms of ERK phosphorylation occurring in advance of nuclear translocation. Serum-starved cells were stimulated with various concentrations of EGF, and the levels of phosphorylated ERK were analysed by western blotting (Fig. 1b; Supplementary Fig. 1). The response data were fitted with the Hill function where the Hill coefficient (*n*_H_) represents the degree of ultrasensitivity, giving *n*_H_=1.2 (Fig. 1c). The ratio of EC_90_ to EC_10_ (EC_90_/EC_10_) that is represented as 81^(1/*n*H)^ is useful to understand the degree of ultrasensitivity of the response^32^. The value of EC_90_/EC_10_ was ∼39 in the present study, indicating that a difference in input signal of more than an order of magnitude was required to switch the system from 10 to 90% maximal output. Therefore, ERK phosphorylation is induced in a graded manner in response to EGF stimulation. Note that the time courses of ERK phosphorylation in response to different EGF concentrations were similar except for the amplitude, suggesting that the result of the dose–response relation was less dependent on the time point at which the response was investigated (Supplementary Fig. 2).

The graded response observed by western blotting may reflect a mixture of ‘on' and ‘off' cells at a single-cell level. To examine this possibility, we stimulated cells with high (50 ng ml^−1^>EC_90_), intermediate (0.3 ng ml^−1^≈EC_50_) and low (0.01 ng ml^−1^<EC_10_) concentrations of EGF and analysed the ERK phosphorylation in individual cells using immunofluorescence. If the graded response observed by western blotting is due to a mixture of ‘on' and ‘off' cells, then the histogram of the immunofluorescence intensity would be bimodal. The results confirmed that the distributions were unimodal rather than bimodal (Fig. 1d), leading to rejection of the above suggestion. These results were also consistent with previous studies that examined EGF-induced ERK phosphorylation in single cells using immunofluorescence and flow cytometry^10^,^11^. Taken together, these observations indicated that EGF stimulation induced ERK phosphorylation in a graded manner.

### Nuclear translocation of ERK behaves like a switch

**Figure 2:**
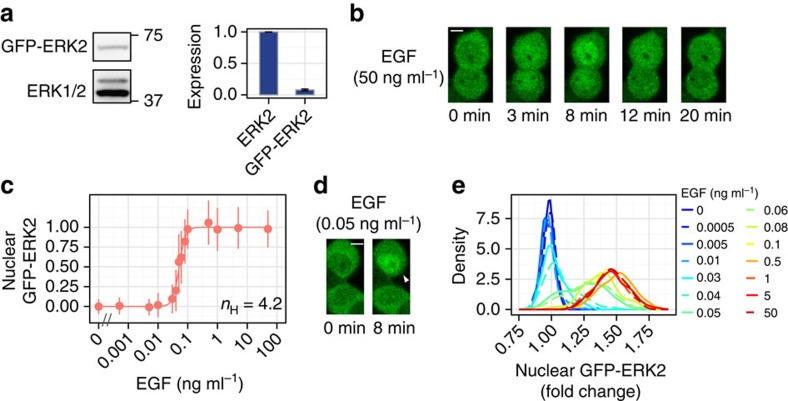
Switch-like response of EGF-induced ERK nuclear translocation. (**a**) The lysate from PC12 cells stably expressing GFP–ERK2 was subjected to western blotting analysis with anti-ERK antibody. The expression level of GFP–ERK2 was much lower than that of endogenous ERK2, which is important to ensure few artefacts in the behaviour or cellular localization of ERK. Error bars represent standard errors of three independent experiments. (**b**) Time-lapse images of EGF-induced (50 ng ml^−1^) GFP–ERK2 nuclear translocation as observed by live-cell imaging using a confocal microscope. Scale bar, 5 μm. (**c**) The levels of nuclear GFP–ERK2 at 8 min after stimulation are shown as a function of EGF concentration with s.d. At least 180 cells were observed in each condition. Hill coefficient was obtained by curve fitting with a Hill function. (**d**) Nuclear translocation of GFP–ERK2 was induced in an all-or-none manner (white arrow) among individual cells upon stimulation with 0.05 ng ml^−1^ of EGF. Scale bar, 5 μm. (**e**) Distributions of nuclear GFP–ERK2 intensities (fold change) at 8 min after EGF stimulation.

We then investigated the dose–response relation of ERK to EGF in terms of nuclear translocation. To visualize the stimulus-induced nuclear translocation of ERK in living cells, we constructed cells stably expressing GFP–ERK2 and histone H2B-mRFP1 as markers for ERK2 localization and the nucleus, respectively. Note that we used a clone expressing a low level of GFP–ERK2 compared with endogenous ERK (Fig. 2a) because overexpression of GFP–ERK2 sometimes causes abnormal ERK2 localization patterns^33^. We also confirmed that there were no differences in the dynamics or dose–response of EGF-induced phosphorylation between GFP–ERK2 and authentic ERK (Supplementary Fig. 3). On EGF stimulation, transient GFP–ERK2 translocation was observed at the single-cell level (Fig. 2b). We also confirmed that the translocation was blocked by inhibition of MEK, a kinase of ERK, indicating that nuclear translocation requires ERK phosphorylation (Supplementary Fig. 4). Then, we stimulated cells with various concentrations of EGF and examined the cellular localization of GFP–ERK2 in thousands of living single cells to investigate the dose–response relation of ERK nuclear translocation to EGF stimulation (Supplementary Fig. 5). To measure the increase in nuclear GFP–ERK2 concentration in individual cells, the intensity of GFP–ERK2 in the nucleus was normalized relative to the value before stimulation^34^, and the dose–response relation was determined. Surprisingly, we found that the response of nuclear translocation was much steeper than that of phosphorylation (Fig. 2c). The response was well fitted by a Hill function with *n*_H_=4.2 (EC_90_/EC_10_=2.8), indicating that the ‘on' and ‘off' states of the system can be switched by approximately threefold differences in input signal. Taken together, these results indicated that ERK nuclear translocation is induced in a switch-like manner.

We further analysed the responses in individual cells. On stimulation with an apparent EC_50_ (∼0.05 ng ml^−1^) of EGF, individual cells typically did not show a ‘half' response but showed an ‘all-or-none' response (Fig. 2d). Indeed, the distribution of nuclear GFP–ERK2 intensity in single cells after EGF stimulation seemed to be bimodal (Fig. 2e). For quantitative assessment of the distribution, we calculated the mutual information between EGF and ERK nuclear translocation. In the context of input–output relation in cellular signalling, the mutual information indicates how many different input states are distinguishable according to a certain output value, or how many output values can be adopted by the input^35^. Here the mutual information was ∼1 bit (Supplementary Fig. 6), suggesting that EGF induces at most two distinct modes of ERK nuclear translocation response in a single cell.

### The graded to switch-like conversion is a general property

**Figure 3:**
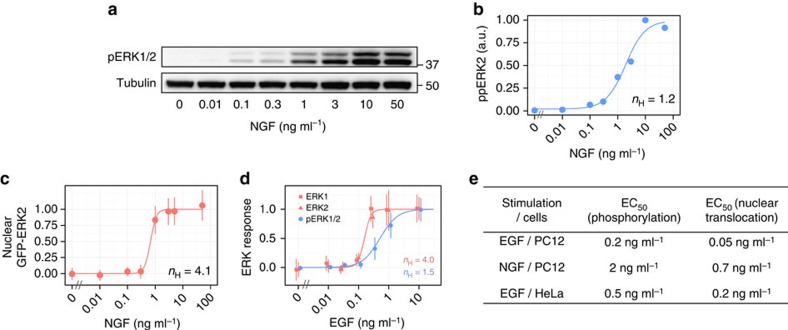
The graded to switch-like conversion is a general property in growth factor-induced ERK signalling. (**a**) Serum-starved PC12 cells were stimulated with the indicated concentrations of NGF for 5 min, and the lysates were subjected to Western blotting analysis with anti-phospho-ERK1/2 antibody. (**b**) The levels of phosphorylated ERK2 (a.u.) were plotted as a function of NGF concentration. (**c**) The levels of nuclear GFP–ERK2 at 9 min after stimulation are shown as a function of NGF concentration with s.d. At least 148 cells were observed in each condition. The Hill coefficient was obtained by curve fitting with a Hill function. (**d**) Serum-starved HeLa cells were stimulated with the indicated concentrations of EGF for 10 min, and then triply immunostained with anti-human ERK1, anti-ERK2 and anti-phospho-ERK1/2 antibodies. Immunofluorescence images are shown in Supplementary Fig. 9. The levels of ERK1/2 phosphorylation (blue) and nuclear/cytoplasmic ratio of ERK1 and ERK2 (red) were plotted as a function of EGF concentration with standard deviations. At least 100 cells were observed in each condition. The Hill coefficients were obtained by curve fitting with a Hill function. (**e**) EC_50_ concentrations of growth factors for stimulus-induced ERK phosphorylation and ERK nuclear translocation response.

We then tested the ERK response in two additional systems, that is, nerve growth factor (NGF) stimulation in PC12 cells and EGF stimulation in HeLa cells, to examine the generality of the graded to switch-like conversion. NGF is known to induce neuronal differentiation in PC12 cells through activation of ERK signalling^36^. In response to NGF stimulation, ERK shows transient and then sustained activation where the initial dynamics are similar to those induced by EGF (Supplementary Fig. 7). Here we focused on the NGF-induced initial activation of ERK and analysed the dose–response relation. As with previous experiments, we stimulated cells with various concentrations of NGF, and analysed the ERK response in terms of phosphorylation and nuclear translocation. The results indicated that NGF also induces ERK phosphorylation and nuclear translocation in graded (*n*_H_=1.2, EC_90_/EC_10_=39) and switch-like (*n*_H_=4.1, EC_90_/EC_10_=2.9) manners, respectively (Fig. 3a–c).

Although we monitored the nuclear translocation of ERK using live-cell imaging with an exogenous GFP–ERK2 probe, it would be informative to also test endogenous ERK behaviours. Therefore, the EGF-induced ERK response was investigated using immunofluorescence with anti-human ERK1, anti-ERK2 and anti-phospho-ERK1/2 antibodies in HeLa cells. Anti-human ERK1 and anti-ERK2 antibodies were not cross-reactive with ERK2 and ERK1, respectively (Supplementary Fig. 8), which allowed analyses of ERK nuclear translocation response at the level of the isoform. Again, we stimulated serum-starved HeLa cells with various concentrations of EGF and analysed ERK phosphorylation and nuclear translocation (Supplementary Fig. 9). As observed in PC12 cells, we confirmed that the phosphorylation was induced in a graded manner (*n*_H_=1.5, EC_90_/EC_10_=19) even in HeLa cells (Fig. 3d, blue). On the other hand, immunofluorescence of anti-human ERK1 and anti-ERK2 antibodies clearly indicated that the nuclear translocation in response to EGF was induced in a switch-like manner (*n*_H_=4.0, EC_90_/EC_10_=3.0) in HeLa cells (Fig. 3d, red).

Thus, we confirmed that graded phosphorylation is converted into switch-like nuclear translocation in at least three different systems, that is, EGF-stimulated PC12 cells, NGF-stimulated PC12 cells and EGF-stimulated HeLa cells. These results indicate that the conversion of graded phosphorylation into switch-like nuclear translocation is a general property of growth factor-induced ERK signalling. Note that the EC_50_ concentration of growth factors for ERK nuclear translocation response was smaller than that for the ERK phosphorylation response, which was observed in all systems examined (Fig. 3e). Therefore, the process of nuclear translocation increases in both steepness of the response (*n*_H_) and sensitivity to the input signal (EC_50_).

### Switch-like nuclear translocation depends on ERK activity

**Figure 4:**
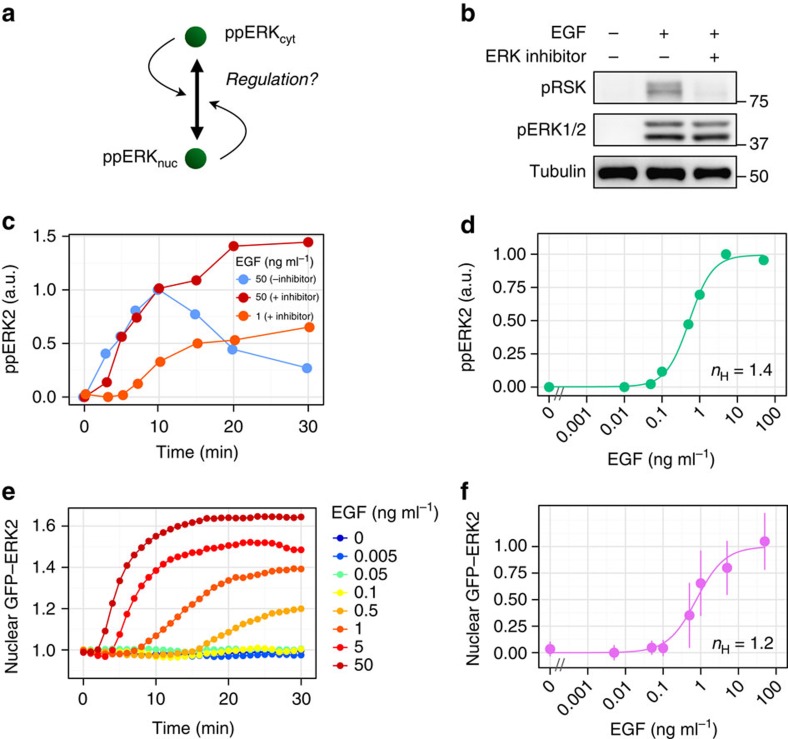
Switch-like nuclear translocation is dependent on ERK kinase activity. (**a**) Schematic representation of possible relationship between ERK activity and nuclear translocation. (**b**) PC12 cells were treated with FR180204 or DMSO and subsequently stimulated with EGF (50 ng ml^−1^) for 10 min. The lysates were subjected to western blotting analysis with the indicated antibodies. (**c**) Time courses of ERK2 phosphorylation (a.u.) induced by EGF stimulation in ERK inhibitor-treated or non-treated PC12 cells. (**d**) The levels of phosphorylated ERK2 (a.u.) at 30 min after EGF stimulation in ERK inhibitor-treated cells were plotted as a function of EGF concentration. The Hill coefficient was obtained by curve fitting with a Hill function. (**e**) Cells were treated with ERK inhibitor and stimulated with the indicated concentrations of EGF, and the time courses of nuclear GFP–ERK2 intensities (fold change) are shown. (**f**) The levels of nuclear GFP–ERK2 at 30 min after stimulation in ERK inhibitor-treated cells are shown as a function of EGF concentration with standard deviations. At least 132 cells were observed in each condition. The Hill coefficient was obtained by curve fitting with a Hill function.

As ERK phosphorylation showed a graded response, whereas nuclear translocation showed a switch-like response, there should be some mechanism(s) for the conversion from a graded to a switch-like response. Although there are several possible mechanisms to generate ultrasensitive responses, one of the most commonly observed is an autoregulatory mechanism that is dependent on its own activity (Fig. 4a). Therefore, we examined whether the switch-like behaviour of nuclear translocation was dependent on ERK activity using the ERK inhibitor FR180204 (ref. 37). Treatment of cells with the ERK inhibitor was confirmed to extensively block the phosphorylation of p90RSK, which is one of the major substrates of ERK (Fig. 4b). Note that the dynamic property of ERK phosphorylation after 10 min was changed by the ERK inhibitor, which showed sustained dynamics for at least 30 min after stimulation (Fig. 4c). This was probably due to inhibition of negative feedback from ERK to the upstream of ERK^38^. Nevertheless, the response of ERK phosphorylation was still graded (*n*_H_=1.4, EC_90_/EC_10_=23) even in ERK inhibitor-treated cells (Fig. 4d). In contrast, ERK nuclear translocation showed a graded rather than switch-like response (*n*_H_=1.2, EC_90_/EC_10_=39) in ERK-inhibitor-treated cells (Fig. 4e,f). Nuclear translocation also showed sustained dynamics similar to phosphorylation, and the initial slope of nuclear import and the level of nuclear accumulation were proportional to the EGF concentration (Fig. 4e). These findings strongly suggested that ERK activity plays a significant role in the switch-like nuclear translocation response. That is, the switch-like response of ERK nuclear translocation is achieved by autoregulatory mechanisms.

### Phosphorylation of nucleoporins regulates ERK translocation

**Figure 5:**
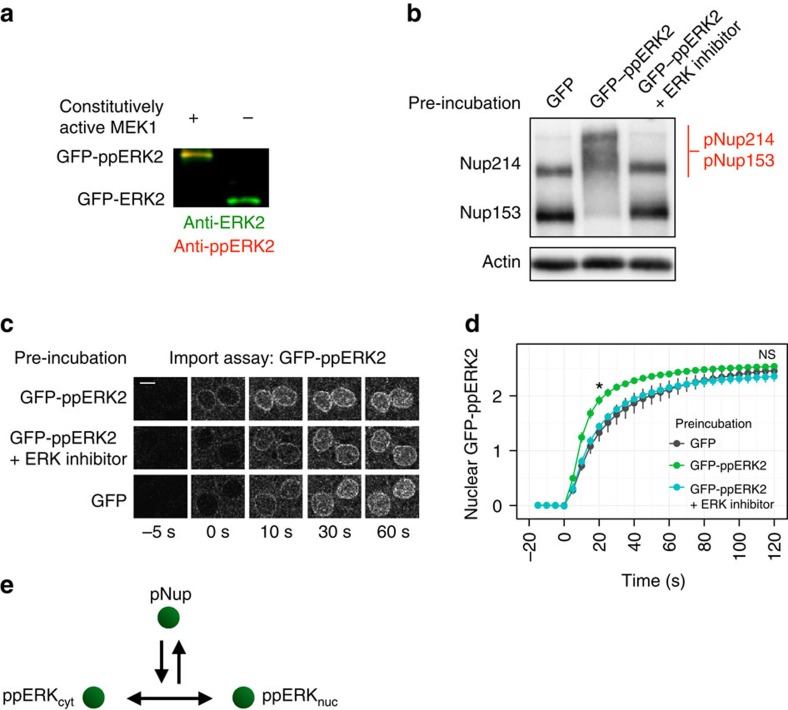
ERK-mediated phosphorylation of nucleoporins enhances ERK nuclear translocation. (**a**) Purification of GFP–ppERK2. *E. coli* was co-transformed with plasmids of GFP–ERK2 and constitutively active MEK1 to obtain phospho-form of GFP–ERK2. Phosphorylation was confirmed by Mn^2+^-Phos-tag SDS-PAGE, followed by immunoblotting with anti-ERK mouse antibody and Alexa Fluor 488-conjugated anti-mouse IgG antibody as a secondary antibody, anti-ppERK2 rabbit antibody and Alexa Fluor 647-conjugated anti-rabbit IgG antibody as a secondary antibody. (**b**) *In vitro* phosphorylation of nucleoporins (Nups) in digitonin-permeabilized cells. Digitonin-permeabilized cells were preincubated with GFP–ppERK2 or GFP (negative control), with ERK inhibitor or DMSO to induce ERK-mediated phosphorylation of Nups. Phosphorylation was confirmed by Mn^2+^-Phos-tag western blotting analysis. (**c**) Nuclear import of GFP–ppERK2 was observed in digitonin-permeabilized cells at a time resolution of 5 s. Scale bar, 5 μm. (**d**) Time courses of GFP-ppERK2 nuclear import were quantified and shown with standard errors of three independent experiments. Student's *t*-test was used to analyse the data at 20 and 120 s. *P<0.05, NS, not significant. (**e**) Schematic representation of nuclear translocation regulation via ERK-mediated phosphorylation of Nups.

The above results with ERK inhibitors raised questions regarding the link between ERK activity and nuclear translocation regulation. Among ERK substrates reported to date^39^, NPC proteins including FG Nups are related to nucleocytoplasmic translocation. Indeed, it has been reported that ERK-mediated phosphorylation of FG Nups affects the nuclear translocation of importin-β (ref. 27). Therefore, one possible scenario for the link between ERK activity and ERK nuclear translocation is that ERK regulates the NPC environment through phosphorylation, which leads to changes in the kinetics of ERK nuclear translocation. To validate this hypothesis, we reconstructed ERK nuclear translocation in digitonin-permeabilized cells. Before the import assay, we incubated digitonin-permeabilized cells with recombinant GFP–ppERK2 (Fig. 5a) to induce ERK-mediated phosphorylation of Nups. We confirmed that at least four of the FG Nups known as ERK substrates^27^,^28^, that is, Nup214, Nup153, Nup62 and Nup50, were phosphorylated in digitonin-permeabilized cells (Fig. 5b; Supplementary Fig. 10). After washing cells with transport buffer, we examined time-lapse images of GFP–ppERK2 nuclear translocation (Fig. 5c). The results showed that GFP–ppERK2 entered the nucleus faster in cells that were preincubated with GFP–ppERK2 than in those preincubated with GFP or GFP–ppERK2 plus ERK inhibitor (Fig. 5d). For example, the nuclear intensity at 20 s was significantly higher in GFP–ppERK2-treated cells (*P*<0.05; *t*-test). The steady-state level (∼120 s) was not significantly different (*P*>0.05; *t*-test), indicating that the effect was bidirectional. Therefore, we found that the ERK-mediated phosphorylation of Nups accelerates nucleocytoplasmic translocation of ERK. This was in contrast with the case of importin-β, the nuclear import of which was reduced, indicating that ERK-mediated phosphorylation of Nups can both up- and downregulate nucleocytoplasmic translocation. Taken together, these observations indicated that ERK nuclear translocation was enhanced by ERK-mediated phosphorylation of Nups, which is one of the molecular mechanisms involved in autoregulation of ERK nuclear translocation (Fig. 5e). Note that the translocation in this assay was carrier-independent because the assay was performed with digitonin-permeabilized cells that lacked cytoplasmic factors. Similarly, there was no cytoplasmic sequestration of ERK by anchor proteins, and therefore the apparent rate of nuclear translocation in the assay was fast compared with the time scale of the stimulus-induced nuclear translocation of ERK in living cells.

### Kinetic modelling of ERK nuclear translocation

**Figure 6:**
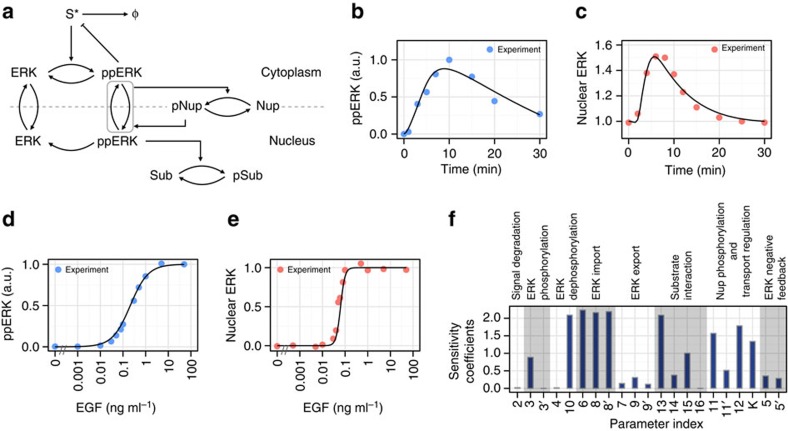
Mathematical modelling of ERK phosphorylation and nuclear translocation. (**a**) Topology scheme of the model. (**b**) Time courses of ppERK and (**c**) nuclear ERK in response to EGF (50 ng ml^−1^) stimulation were numerically simulated with the model (solid lines). Points represent experimental data. (**d**) Dose–response relation of EGF and ppERK or (**e**) nuclear ERK. Points and solid lines indicate experimental and simulation data, respectively. (**f**) Sensitivity analysis for the Hill coefficient of ERK nuclear translocation response. Sensitivity coefficients were calculated by making 1% changes in parameters and absolute values are shown.

The results outlined above are strongly suggestive of a link between ERK activity and ERK nuclear translocation; however, it is not clear whether the ERK-mediated regulation of its own nuclear translocation is adequate to reproduce experimentally observed data, including the conversion from a graded to a switch-like ERK response. To investigate this question, we constructed an ordinary differential equation model of ERK phosphorylation and nuclear translocation accompanied with translocation regulation via Nups. The basic structure of the model was derived from the previous study, which explained the time courses of both phosphorylation and nuclear translocation^17^. The model was updated by integrating our results of ERK-mediated Nup phosphorylation and translocation regulation (Fig. 6a; Supplementary Note 1). The model parameters were fitted to the experimental data by optimization (Supplementary Tables 1–3). Consequently, the model well reproduced the time course of both ERK phosphorylation and nuclear translocation (Fig. 6b,c). In addition, the model successfully realized conversion of graded phosphorylation into switch-like nuclear translocation (Fig. 6d,e). Note that we could not reproduce the observed data by the model without Nups. To understand the processes responsible for the switch-like response, we calculated sensitivity coefficients for the Hill coefficient of ERK nuclear translocation response by making 1% changes in model parameters. The results indicated that not only parameters that were directly related to nuclear ERK but also those related to Nups showed large sensitivity coefficients (Fig. 6f). These results strongly suggest a significant role of the newly introduced processes, autoregulatory mechanisms for ERK translocation in the conversion from a graded to a switch-like ERK response.

### Depletion of Nup153 affects the ERK nuclear translocation

**Figure 7:**
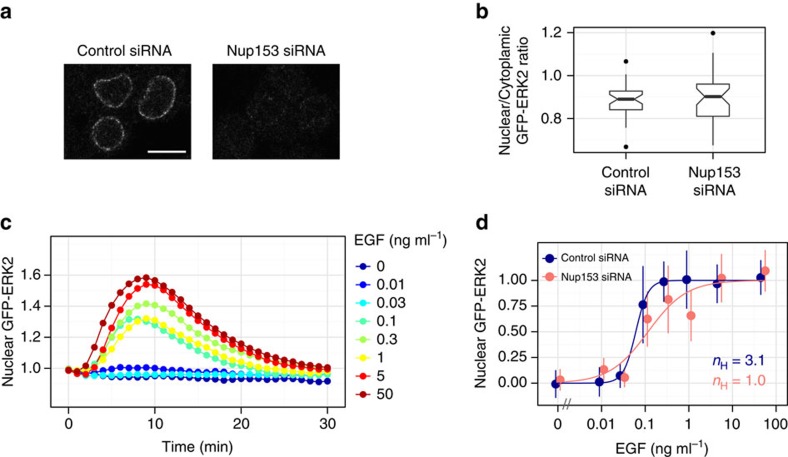
Depletion of Nup153 affects the ERK nuclear translocation response. (**a**) Cells were transfected with Nup153 siRNA or control siRNA duplexes, and immunostained with anti-Nup153 antibody at 64 h after transfection. Scale bar, 10 μm. (**b**) The distributions of nuclear/cytoplasmic ratio of GFP–ERK2 in Nup153-depleted or non-depleted cells are shown as box plots. No significant differences were observed (*P*>0.05; *t*-test). (**c**) Time courses of mean nuclear GFP–ERK2 intensities (fold change) in Nup153-depleted cells. Cells were stimulated with the indicated concentrations of EGF at 64 h after Nup153 siRNA transfection. Immediately after the live imaging experiments, cells were fixed, immunostained with anti-Nup153 antibody and the Nup153-depleted cells were used for the analyses. At least 34 cells from 4 independent fields of view were analysed in each condition. (**d**) The levels of nuclear GFP–ERK2 at 9 min after stimulation in Nup153-depleted or non-depleted cells are shown as a function of EGF concentration with standard deviations. The Hill coefficient was obtained by curve fitting with a Hill function.

The results of *in vitro* and *in silico* analyses in the present study suggested a correlation between Nup phosphorylation and ERK nuclear translocation. However, it remains unclear if Nups modulate ERK behaviours in living cells. Therefore, we investigated ERK nuclear translocation with depletion of Nup153 (Fig. 7a), one of the relevant Nups that is most effectively phosphorylated by ERK^27^. Knockdown of Nup153 did not cause any abnormal ERK2 localization patterns before stimulation (Fig. 7b, *P*>0.05; *t*-test). As with previous experiments, cells were stimulated with various concentrations of EGF and the nuclear translocation response of GFP–ERK2 was investigated (Fig. 7c). The results indicated that the steepness of the EGF-induced ERK nuclear translocation response was more gradual in Nup153-depleted cells than in non-depleted cells (Fig. 7d), suggesting a contribution of Nup153 to regulation of the ERK nuclear translocation response.

## Discussion

ERK MAP kinase signalling is an important master regulator of the cell fate decision in various mammalian cells. This system was therefore expected to behave like a switch, while the output of the system defined by the amount of phosphorylation of ERK has been shown to display a linear response, which raises questions regarding how such a linear system can produce completely different cell fates in individual cells. Here we presented evidence that, rather than phosphorylation itself, subsequent nuclear translocation accompanied by autoregulatory mechanisms acts as a switch in ERK signalling.

The ultrasensitive switch-like response of ERK signalling could be significant for several reasons. First, switch-like behaviour that filters out noise or markedly low-level extracellular signals could be important to avoid erroneous responses. Otherwise, ERK would be induced even without extracellular signals, which would probably lead to various dysfunctions in cell physiology^40^. Second, the ultrasensitivity of the system eliminates the ambiguity of the signal. This is reasonable as ERK regulates the cell fate decision, including proliferation, differentiation, survival and death, which are essentially all-or-none biological phenomena. Indeed, a pulse-like increase in ERK activity in the nucleus was observed using FRET biosensors in proliferating cells^41^. They also revealed that the frequency of the pulse, but not the amplitude, was significant for ERK-dependent cell proliferation. Therefore, the reduction of ambiguity in ERK signalling would be reasonable for cells.

The switch-like nuclear translocation, but not phosphorylation, may enable different ERK activities in the cytoplasm and nucleus; that is, cytoplasmic substrates would be phosphorylated in a graded manner, while phosphorylation of nuclear substrates would be regulated in a switch-like manner. Indeed, it has been reported that phosphorylation of RSK1, one of the major cytoplasmic targets of ERK, shows a graded response, while phosphorylation of c-Fos that is localized to the nucleus is more sensitive than that of RSK1 (ref. 10), although the underlying mechanism was unclear. Thus, the non-linear property of ERK nuclear translocation may be the basis for the distinct ERK activities in each subcellular location. Consistent with this suggestion, regulation of ERK activity at the level of subcellular localization has recently been reported to be tightly associated with the cell fate decision in myogenesis where cytoplasmic and nuclear ERK induce differentiation and proliferation, respectively^42^.

The regulation of nuclear translocation in ERK signalling could be understood in terms of the increase in sensitivity to growth factors because the EC_50_ for the nuclear translocation response was lower than that for phosphorylation (Fig. 3e). That is, at most less than half-maximal level of ERK phosphorylation is sufficient to fully evoke stimulus-induced nuclear translocation. Recently, the relationship between ERK phosphorylation and cell proliferation was investigated quantitatively, and the results indicated that the basal level of ERK phosphorylation was ∼10%, which reaches only ∼30% even at the peak^41^. Therefore, cells use only 20% changes in the level of ERK phosphorylation under physiological conditions, but this would be sufficient to effectively transfer the extracellular information into the nucleus in a switch-like manner.

In the present study, we proposed that ERK-mediated phosphorylation of Nups is one of the possible autoregulatory mechanisms. Although only a few proteins, including NPC proteins, are known as substrates of ERK that seem to be related directly to nucleocytoplasmic translocation, it remains possible that there are also other underlying mechanisms. As shown in our import assay as well as in previous studies, ERK is able to enter the nucleus without a carrier or energy. In contrast, a carrier-dependent pathway that depends on Imp7 has also been reported, which involves phosphorylation on the Ser residues at an SPS (Ser244, Pro245 and Ser246) motif in the kinase insert of ERK^43^. The SPS motif lies within an ERK consensus phosphorylation site and Ser244 is indeed phosphorylated by ERK^25^. Therefore, the autophosphorylation may act as an autoregulatory mechanism for nuclear translocation of ERK, although Casein kinase 2 mostly mediates SPS phosphorylation *in vivo*^25^. In addition, the subcellular localization of ERK is regulated by cytoplasmic sequestration through interactions with several anchor proteins, such as MEK, Sef and PEA-15 (refs 44, 45, 46). If the sequestration by the anchor proteins is under control of ERK activity, then this would be another mechanism for the autoregulation of ERK translocation.

Non-linear behaviours, including ultrasensitivity, oscillation and bistability, are the basis of the higher order function of biological systems. The complex nature of the ERK MAP kinase phosphorylation cascade has been studied extensively, while subsequent nuclear translocation has often been neglected. Nevertheless, our study demonstrated that ERK phosphorylation is likely to show a mostly linear response, at least in mammalian cells, whereas nuclear translocation is a highly regulated process that behaves like a switch. Thus, our study shed light on the importance of not only the phosphorylation cascade but also nuclear translocation, which characterizes the system behaviour in the ERK signalling pathway.

## Methods

### Plasmid preparation and reagents

The complementary DNA of human ERK2 was inserted into pEGFP-C2 vector with a monomeric A206K mutation (GFP–ERK2). A fragment of GFP–ERK2 was introduced into pGEX2T vector, and then an N-terminal PreScission site and C-terminal His_8_-tag were introduced (pGEX–GFP–ERK2–His). The plasmid H2B-mRFP1 was provided by Dr H. Kimura (Tokyo Institute of Technology)^47^,^48^. A gene encoding a constitutively active form of human MEK1 (S218/222E, Δ32-51) fused to Venus with 8 × Gly linker was introduced into pACYC184 vector (pACYC184–Venus–MEK1 (S218/222E, Δ32-51))^49^. EGF was purchased from PeproTech. The MEK inhibitor, U0126, was purchased from Promega. The ERK inhibitor, FR180204, was purchased from Santa Cruz. Phos-tag acrylamide was purchased from Wako. Digitonin was purchased from Calbiochem. Anti-ERK1/2 (L34F12 and 137F5), anti-phospho-ERK1/2 (E10 and D13.14.4E), anti-phospho-p90RSK (D3H11) and anti-alpha-tubulin (DM1A) antibodies were purchased from Cell Signaling Technology. Anti-human ERK1 (Y72) antibody was purchased from Abcam. Anti-ERK2 (D-2) antibody was purchased from Santa Cruz. Anti-actin IgM (clone JLA20) antibody was purchased from the Developmental Studies Hybridoma Bank. Anti-Nup153 (7A8) antibody was purchased from Progen. QE5 anti-Nup153 antibody that also recognizes Nup214 and Nup62 was purchased from Covance. Rabbit polyclonal anti-Nup50 antibody was generated by immunizing bacterially expressed full-length mouse Nup50 protein and affinity-purified by adsorption to antigen-coupled Affi-Gel 15 (Bio-Rad)^27^. The linearity of western blotting analysis was confirmed by serial dilution of the lysates, followed by immunoblotting with anti-phospho-ERK1/2 antibody. Phos-tag western blotting was performed in gels containing 7.5% acrylamide, 50 μM MnCl_2_ and 25 μM Phos-tag, followed by immunoblotting.

### Cell culture

Rat PC12 pheochromocytoma cells were purchased from RIKEN Cell Bank. Cells stably expressing GFP–ERK2 and H2B-mRFP1 were generated by transfection with Effectene (Qiagen). Cells were maintained in DMEM supplemented with 10% horse serum and 5% fetal bovine serum. A selected clone of HeLa cells, in which chromosome segregation errors are less frequent, was provided by M. Ohsugi (University of Tokyo, Japan) and maintained in DMEM supplemented with 10% fetal bovine serum^50^. For microscopy experiments, cells were plated on poly-L-lysine-coated coverslips or glass-bottomed dishes, cultured for 12 h and then serum-starved for at least 16 h in DMEM without phenol red supplemented with 1% bovine serum albumin (BSA; DMEM–BSA).

### Immunofluorescence

Cells were plated on poly-L-lysine-coated coverslips or gridded glass-bottomed dishes, serum starved and stimulated with the indicated concentrations of EGF. Cells were fixed with 4% paraformaldehyde for 10 min at room temperature, permeabilized in 0.2% Triton X-100 for 10 min at room temperature or 100% methanol for 10 min at –20 °C and then blocked with 1% BSA for 30 min at room temperature. Cells were then incubated cells primary antibodies (anti-Nup153 (7A8, dilution 1:10), anti-ERK1 (Y72, 1:200), anti-ERK2 (D-2, 1:100), anti-phospho-ERK1/2 (E10, 1:200) antibodies) for 1–2 h at RT, followed by secondary antibodies (Alexa Fluor 647 anti-mouse IgG (1:500), Alexa Fluor 488 anti-rabbit IgG (1:1,000), Alexa Fluor 555 anti-mouse IgG2b (1:1,000), Alexa Fluor 647 anti-mouse IgG1 (1:1,000) antibodies) for 1 h at room temperature.

### Live cell imaging

Cells were plated on poly-L-lysine-coated glass bottomed dishes and serum starved. Before microscopy experiments the medium was exchanged to DMEM–BSA containing 10 mM PIPES (pH 7.4). Intracellular distributions of GFP–ERK2 and H2B-mRFP1 proteins were observed using a scanning confocal microscope with a 60 × /1.49NA oil immersion objective. Cells were stimulated with the indicated concentrations of EGF or NGF on the microscope and time-lapse movies were obtained at a time resolution of 1 min. For the experiments using inhibitors, cells were treated with 10 μM U0126 or 60 μM FR180204 for 30 min before growth factor stimulation.

### Purification of recombinant proteins

The plasmid pGEX–GFP–ERK2–His was transformed into *E. coli* Rosetta, grown in LB medium and expression was induced for 12 h at 20 °C by the addition of 0.1 mM IPTG. For preparation of GFP–ppERK2–His, *E. coli* BL21(DE3) was co-transformed with pGEX–GFP–ERK2-His and pACYC184–Venus–MEK1 (S218/222E, Δ32-51), grown in 2 × YT medium and expression was induced for 12 h at 20 °C by the addition of 0.1 mM IPTG. Before collecting, cells were shaken for 1–3 h at 37 °C to enhance ERK2 phosphorylation. Phosphorylation of GFP–ERK2 was confirmed by Phos-tag western blotting. Protein extracts were incubated with Glutathione Sepharose 4B and GST-tag fused to recombinant proteins was cleaved by PreScission protease. Recombinant proteins were further purified with cOmplete His-tag Purification Resin (Roche) to concentrate full-length GFP–ERK2 protein. Purified proteins were dialysed against 20 mM HEPES (pH 7.3), 110 mM KOAc, 1 mM EGTA and 2 mM DTT.

### *In vitro* import assay

Cells were plated on microslides (ibidi) and serum starved. For preparation of digitonin-permeabilized cells, we incubate cells with 50 μg ml^−1^ digitonin in transport buffer (20 mM HEPES-KOH (pH 7.3), 110 mM KOAc, 2 mM MgOAc, 5 mM NaOAc, 0.5 mM EGTA, 2 mM DTT, 1 × PhosSTOP phosphatase inhibitor cocktail, and 1 × complete mini EDTA-free protease inhibitors) for 5 min at 4 °C. For *in vitro* phosphorylation of nucleoporins by ERK, digitonin-permeabilized cells were incubated with transport buffer containing 1 mM ATP, 15 mM MgCl_2_, and 500 nM GFP or 500 nM GFP–ppERK2 with or without 60 μM FR180204 for 20 min at room temperature. After washing the cells with transport buffer, we added transport buffer containing 1 mM ATP, 15 mM MgCl_2_ and 500 nM GFP–ppERK2 on the microscope, and time-lapse movies were obtained at a time resolution of 5 s.

### Kinetic modelling of ERK nuclear translocation

Equations and parameters are listed in Supplementary Tables. Ordinary differential equations were numerically computed using the routines implemented in the scipy.integrate package (http://docs.scipy.org/doc/scipy/reference/integrate.html). Parameter fitting to our experimental data were carried out based on optimization routines implemented in the scipy.optimize package (http://docs.scipy.org/doc/scipy/reference/optimize.html). Sensitivity coefficient *s*_*i*_ is defined as 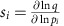, where *q* is a target function and *p*_*i*_ is the *i*th parameter. In this study, the target function was the Hill coefficient of the dose–response relation of ERK nuclear translocation. The sensitivity coefficients were calculated by making 1% changes in each model parameter.

### RNA interference

Stealth Select RNAi oligonucleotide against rat Nup153 (rat Nup153 siRNA-#1 (sense), 5′-ACUUCAGUUUCUGGUCGCAAGAUAA-3′) and Stealth RNAi negative control duplex-#2 with medium GC content were purchased from Invitrogen. Cells were transfected with 30 nM siRNA using Lipofectamine 3000 reagent (Invitrogen) according to the manufacturer's protocol and observed 64 h after transfection. The fraction of Nup153-depleted cells was approximately 50–90%. Therefore, the cells were fixed immediately after the live cell imaging experiments followed by immunofluorescence using anti-Nup153 antibody to identify cells in which Nup153 was depleted.

### Data analysis

To analyse the nuclear translocation of GFP–ERK2, nuclei were detected from H2B-mRFP1 images and nuclear intensities of GFP–ERK2 were measured at each time point using custom-made plug-ins of Fiji/ImageJ (fiji.sc/Fiji). For live cell imaging data analysis, nuclear intensities of GFP–ERK2 were normalized by the mean values before stimulation to obtain fold change increase in stimulus-induced ERK nuclear translocation. For *in vitro* import assay, nuclear intensities were normalized by the intensity of the bulk solution.

The mutual information is defined as *I*(*R*;*S*)=*H*(*R*)−*H*(*R*|*S*)=*H*(*S*)−*H*(*S*|*R*), where 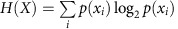 represents the entropy. Direct computation of the mutual information is often biased because of the finite (limited) data. Therefore, unbiased value of the mutual information was estimated using an approximated linear relation defined as 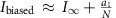, where *N* is the total number of samples, *a*_1_ is a coefficient that depends on *R* and *S*, and *I*_biased_ and *I*_∞_ are the biased and unbiased estimate of the mutual information, respectively^35^. The linear function was estimated by changing *N* from 60 to 100% of the total number of data using jackknife sampling, to obtain the unbiased estimate of the mutual information^35^.

## Additional information

**How to cite this article:** Shindo, Y. *et al.* Conversion of graded phosphorylation into switch-like nuclear translocation via autoregulatory mechanisms in ERK signalling. *Nat. Commun.* 7:10485 doi: 10.1038/ncomms10485 (2016).

## Supplementary Material

Supplementary Information Supplementary Figures 1-10, Supplementary Tables 1-3, Supplementary Note 1 and Supplementary References
