# Supplementary material for: Conversion of graded phosphorylation into switch-like nuclear translocation via autoregulatory mechanisms in ERK signalling
Source: Nat Commun. 2016 Jan 20;7:10485. doi: 10.1038/ncomms10485 (PMC4736105; doi:10.1038/ncomms10485)
Supplement: Supplementary Information — Supplementary Figures 1-10, Supplementary Tables 1-3, Supplementary Note 1 and Supplementary References [file ncomms10485-s1.pdf]

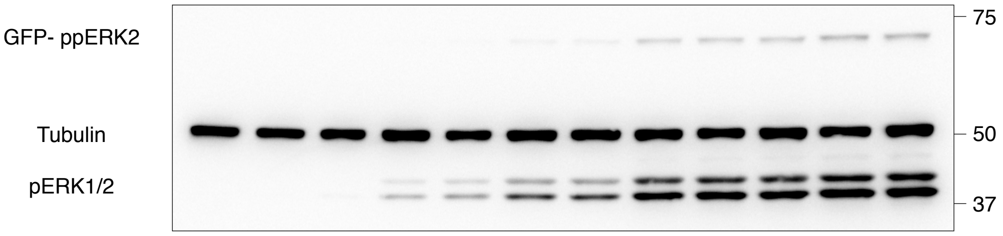

Supplementary Figure 1 Full scan of blots in Fig. 1C.

See the legend of Fig. 1C for details.

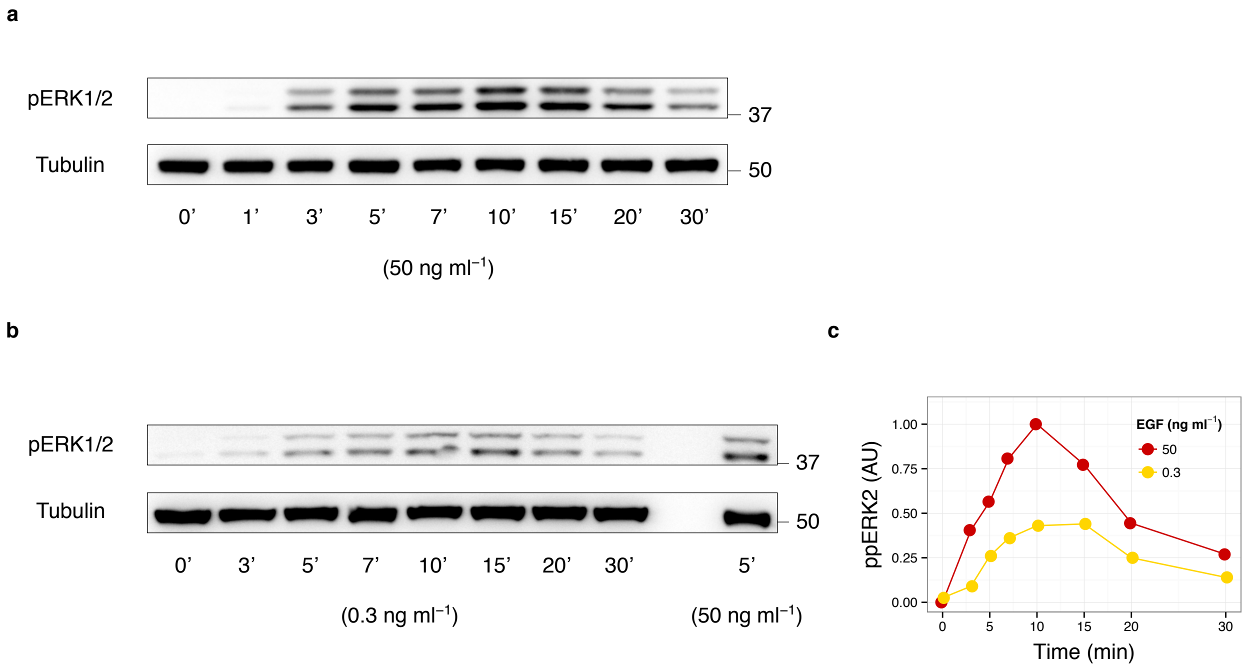

Supplementary Figure 2 Time courses of EGF-induced ERK phosphorylation.

(**a** – **c**) The lysates from EGF-treated (50 or 0.3 ng ml^{-1}) PC12 cells were subjected to Western blotting analysis with anti-phospho-ERK1/2 antibody at the indicated times.

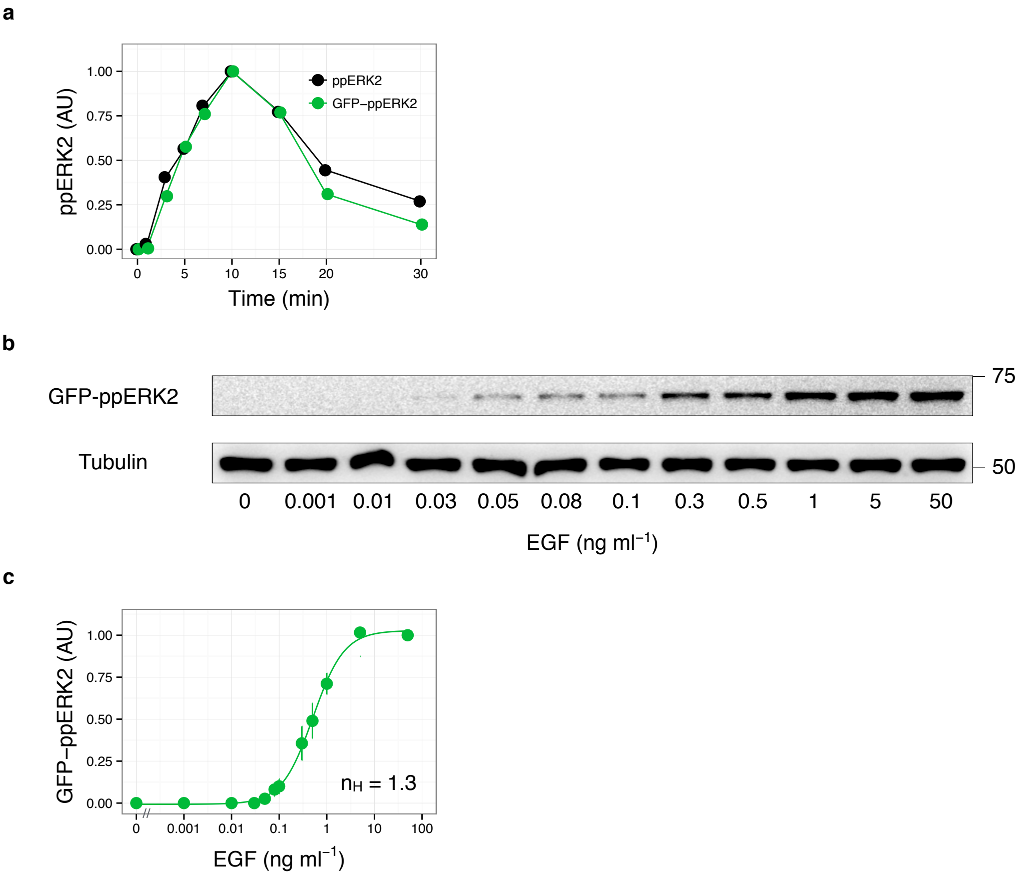

Supplementary Figure 3 Time course and dose–response of exogenous GFP-ERK2.

(**a**) Time course of EGF-induced (50 ng ml^{-1}) phosphorylation of exogenous GFP-ERK2 and endogenous ERK2. (**b, c**) The levels of GFP-ERK2 phosphorylation (AU) were plotted as a function of EGF concentration with standard errors of three independent experiments. Hill coefficient was obtained by curve fitting with a Hill function.

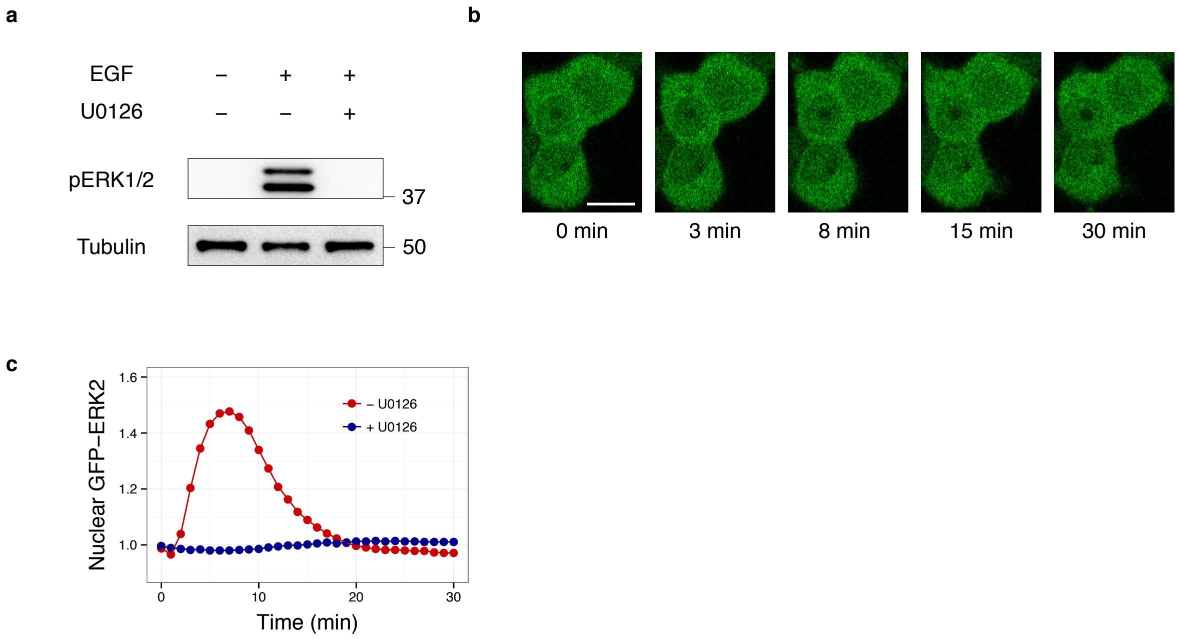

Supplementary Figure 4 U0126 inhibits ERK phosphorylation and nuclear translocation.

(**a**) PC12 cells were treated with 10 μM U0126 or DMSO for 30 min and subsequently stimulated with EGF (50 ng ml^{-1}). The lysates were analysed by Western blotting with anti-phospho- ERK1/2 antibody. (**b**) U0126-treated PC12 cells stably expressing GFP-ERK2 were stimulated with EGF (50 ng ml^{-1}) and time-lapse images were obtained at a time resolution of 1 min. Scale bar, 10 μm. (**c**) Time courses of mean nuclear GFP-ERK2 intensities (fold change) in U0126-treated (410 cells) or non-treated cells (220 cells).

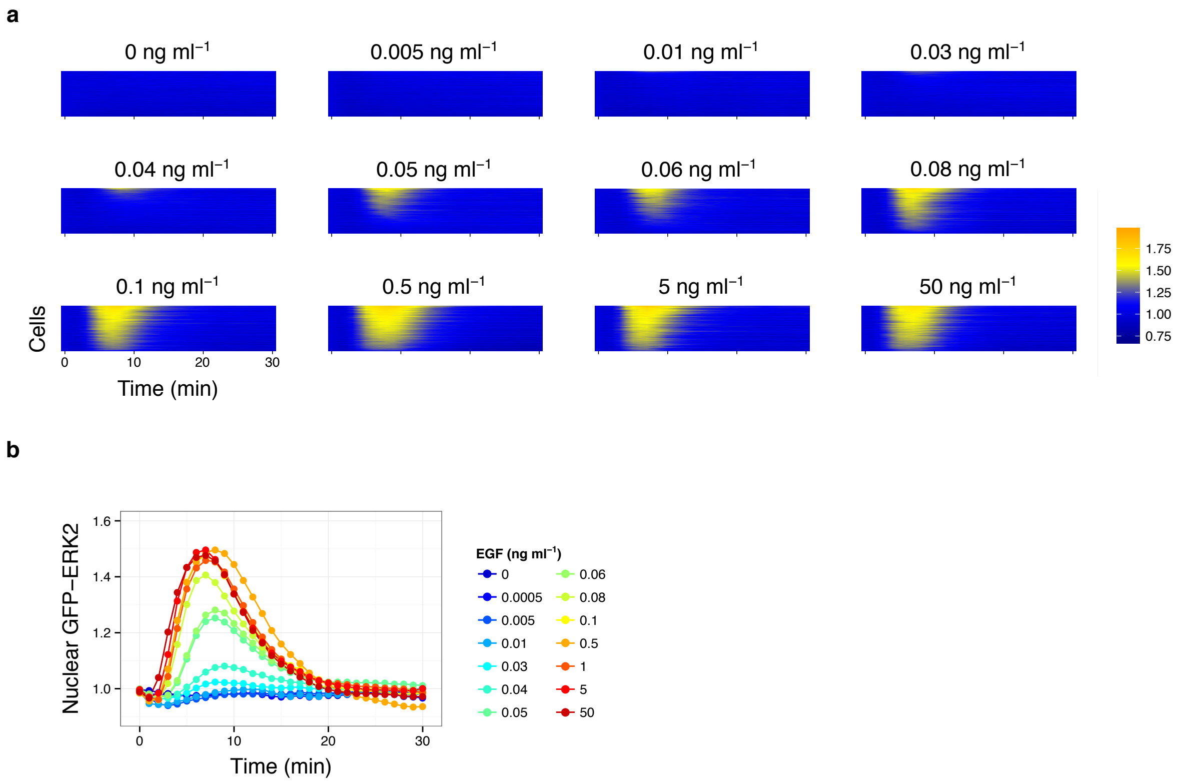

Supplementary Figure 5 EGF-induced nuclear translocation dynamics of GFP-ERK2.

(**a**) Cells were stimulated with the indicated concentration of EGF and time-lapse images were obtained at a time resolution of 1 min. At least 180 cells were observed in each condition and data for single cells (*y*-axis) are shown in the figures. Nuclear intensities of GFP-ERK2 were normalised by the value before stimulation to obtain the fold change increase in nuclear ERK2 concentration. (**b**) Averages of nuclear GFP-ERK2 intensities (fold change) in response to stimulation with the indicated concentrations of EGF.

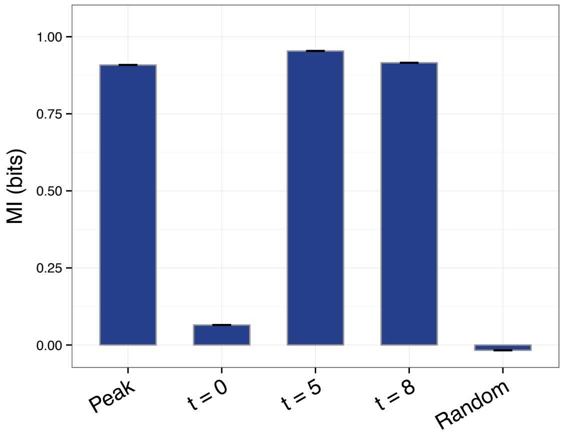

Supplementary Figure 6 Mutual information of nuclear GFP-ERK2 and EGF concentration.

In the context of input–output relation in cellular signalling, the mutual information indicates how many different input states are distinguishable according to a certain output value. From the symmetry of the formula, the mutual information also indicates how many output values can be adopted by the input. Here, the mutual information was approximately 1 bit (0.95 bits), suggesting that EGF induces two distinct modes of ERK nuclear translocation response.

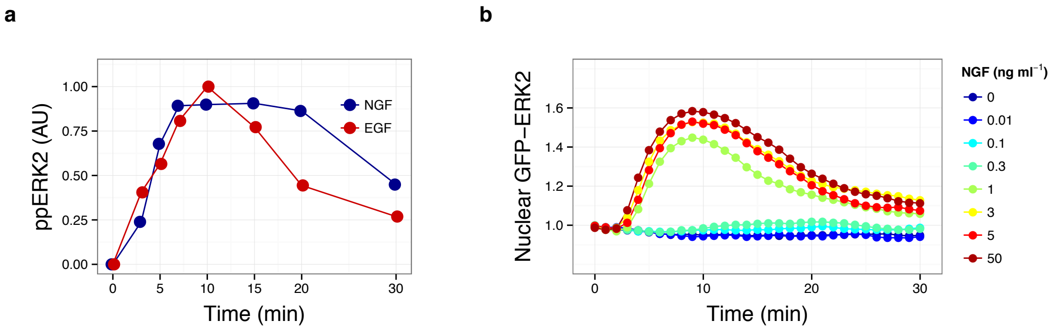

Supplementary Figure 7 Time courses of NGF-induced ERK phosphorylation and nuclear translocation.

(**a**) The lysates from NGF-treated (50 ng ml^{-1}) PC12 cells were analysed by Western blotting with anti-phospho-ERK1/2 antibody at the indicated times, and shown with the time course induced by 50 ng ml^{-1} EGF (duplicated from Supplementary Fig. 2). (**b**) Averages of nuclear GFP-ERK2 intensities (fold change) in response to stimulation with the indicated concentrations of NGF.

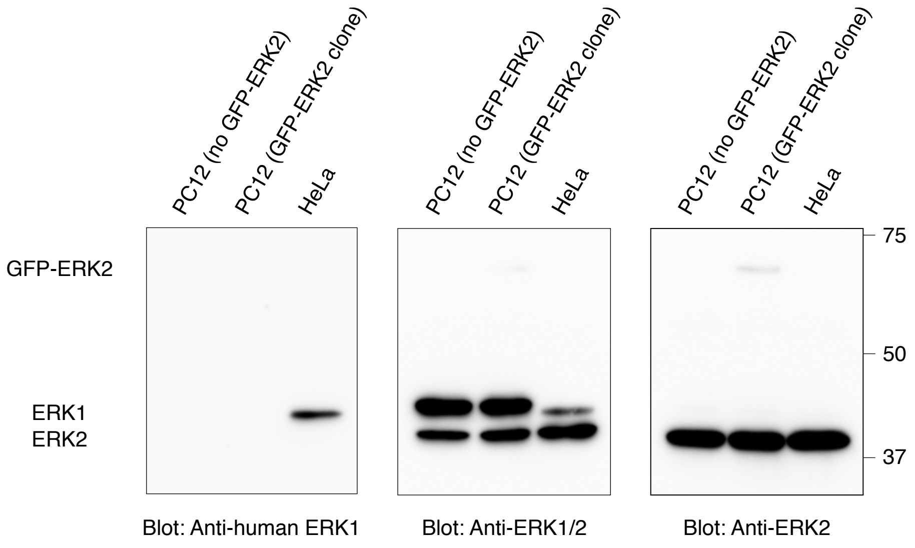

Supplementary Figure 8 Confirmation of the specificity of anti-human ERK1 and anti-ERK2 antibodies.

The lysates from PC12 cells, PC12 cells stably expressing GFP-ERK2, and HeLa cells were subjected to Western blotting analysis with anti-human ERK1 antibody (left), anti-ERK1/2 antibody (middle) and anti-ERK2 antibody (right). Note that no anti-human ERK1 signal was observed in the lysate from PC12 cells derived from rats.

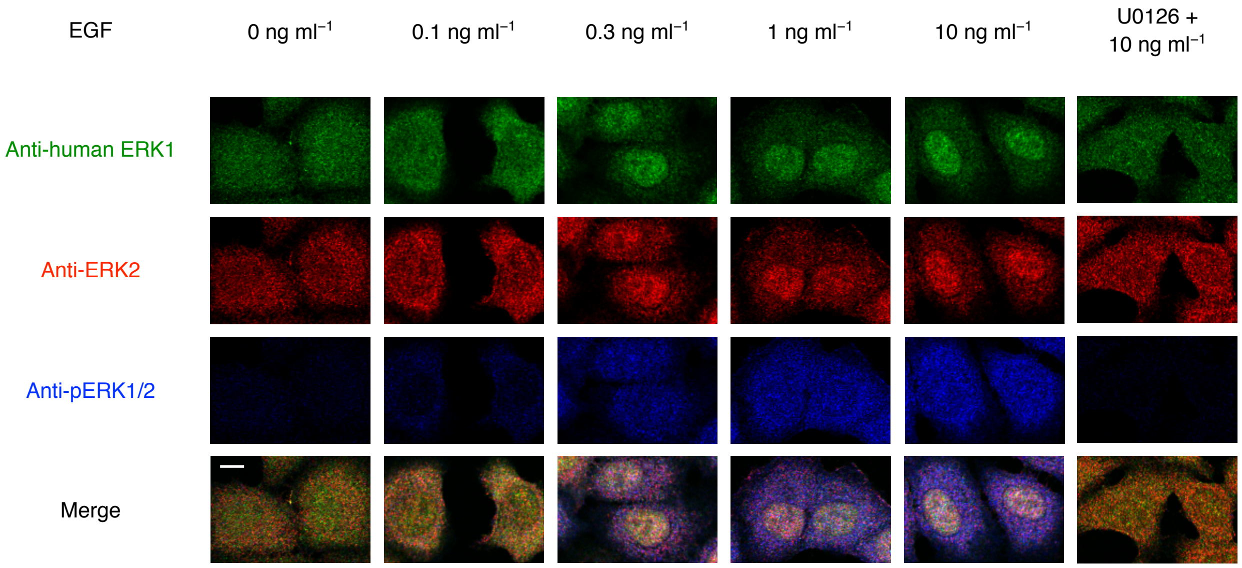

Supplementary Figure 9 EGF-induced ERK response in HeLa cells.

Serum-starved HeLa cells were stimulated with the indicated concentrations of EGF for 10 min, and triply immunostained with anti-human ERK1, anti-ERK2 and anti-phospho-ERK1/2 antibodies. Scale bar, 10 μm.

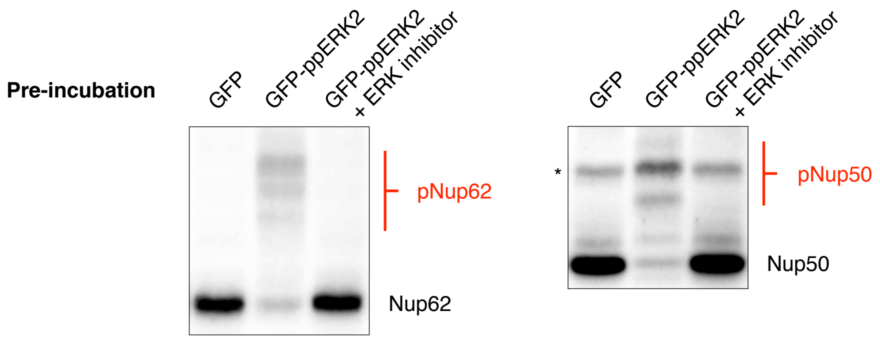

Supplementary Figure 10 Confirmation of ERK-mediated phosphorylation of Nup62 and Nup50 in digitonin-permeabilised cells.

Mn^{2+}-Phos-tag SDS-PAGE, followed by immunoblotting analyses of Nup62 and Nup50. Lysates were obtained as described in Fig. 4b. The asterisk indicates non-specific bands.

Supplementary Table 1 Rate equations of the model.

**Reaction**	**Description**
$v1 = k_1\, S$	Input signal
$v2 = k_2\, S^*$	Decay of signal
$v3 = k_3\, S^* \dfrac{ERK_{cyt}}{k_3' + ERK_{cyt}}$	ERK phosphorylation
$v4 = k_4\, ppERK_{cyt}$	ERK dephosphorylation in the cytoplasm
$v5 = k_5 \dfrac{ppERK_{cyt}}{k_5' + ppERK_{cyt}}$	Negative feedback to signal (Sos)
$v6 = k_6\, ERK_{cyt}$	ERK nuclear import
$v7 = k_7\, ERK_{nuc}$	ERK nuclear export
$v8 = k_8 \dfrac{ppERK_{cyt}}{k_8' + ppERK_{cyt}}(1 + K\, pNup)$	ppERK nuclear import
$v9 = k_9 \dfrac{ppERK_{nuc}}{k_9' + ppERK_{nuc}}(1 + K\, pNup)$	ppERK nuclear export
$v10 = k_{10}\, ppERK_{nuc}$	ERK dephosphorylation in the nucleus
$v11 = k_{11}\, (v8 + v9) \dfrac{Nup}{k_{11}' + Nup}$	Nup phosphorylation
$v12 = k_{12}\, pNup$	Nup dephosphorylation
$v13 = k_{13}\, ppERK_{nuc}\, Sub$	Association of ppERK and substrate
$v14 = k_{14}\, ppERK\_Sub$	Dissociation of ppERK–substrate complex
$v15 = k_{15}\, ppERK\_Sub$	Substrate phosphorylation
$v16 = k_{16}\, pSub$	Substrate dephosphorylation

Supplementary Table 2 Equations and initial conditions.

**Species**	$\boldsymbol{d/dt} =$	**Initial value**[1]
S	$-v1$	$S_0 = EGF/(k_0' + EGF)$
S^*	$v1 - v2 - v5$	0
ERK_{cyt}	$-v3 + v4 - v6 + v7$	480,000[2]
ERK_{nuc}	$v6 - v7 - v10$	96,000[2]
$ppERK_{cyt}$	$v3 - v4 - v8 + v9$	0
$ppERK_{nuc}$	$v8 - v9 - v10 - v14 + v15 + v16$	0
Nup	$-v11 + v12$	5,000[3]
$pNup$	$v11 - v12$	0
Sub	$-v13 + v14 + v16$	96,000[4]
$ppERK\_Sub$	$v13 - v14 - v15$	0
$pSub$	$v15 - v16$	0

[1] Assuming that the cell volume was 1 pl[1]. [2] Total number of ERK molecules was assumed to be 576,000 (~0.96 μM[1]). [3] Assumed based on the literature[2,3]. [4] Assumption (relatively determined by parameter fitting).

Supplementary Table 3 Parameter values.

Parameter	Value[1]
k_0'	0.198 [ng ml^{-1}]
k_1	9.21e-2
k_2	5.90e-2
k_3	8.23e5 [c min^{-1}]
k_3'	9.30e2
k_4	1.01
k_5	2.03e-2
k_5'	3.18e4
k_6	0.18[2]
k_7	0.9[2]
k_8	6.23e4
k_8'	3.47e6
k_9	4.30e4
k_9'	1.41e4
K	2.43e-02 [dimensionless]
k_{10}	3.55e1
k_{11}	1.95 [dimensionless]
k_{11}'	2.46e3
k_{12}	1.60e1
k_{13}	1.12e-3
k_{14}	0.120
k_{15}	0.184
k_{16}	2.86e-6

[1] All units are shown in terms of number of molecules and minute except where otherwise explicitly indicated. c: arbitrary unit of concentration. [2] Assumed based on the literature[4,5].

Supplementary Note 1 Kinetic modelling of ERK phosphorylation and nuclear translocation.

The model is described in the following scheme.

1) A transient input signal is induced in a stimulus-dependent manner and initiates the phosphorylation of cytoplasmic ERK.
2) Cytoplasmic ppERK suppresses the input signals (negative feedback to Sos)
3) ERK and ppERK undergo nucleocytoplasmic shuttling.
4) Nup is phosphorylated by ppERK in proportion to the rate of nucleocytoplasmic shutting of ppERK.
5) Nuclear ppERK binds nuclear substrates and catalyses phosphorylation.
6) Phosphorylated ERK, Nup and substrates are dephosphorylated.

The model did not explicitly include active transport for several reasons as follows. 1) ERK can enter the nucleus without transport carriers or energy[6,7]. 2) Active transport of ERK presumably requires dimerisation, but the rate of transport does not seem to be different between the wild-type and dimerisation-deficient mutant in living cells[8]. 3) The nuclear translocation model with active transport does not improve the fit to the data[9].

For the process of ERK-mediated phosphorylation of Nup, we assumed that ppERK both entering and exiting the nucleus is able to catalyse phosphorylation. Furthermore, the probability that Nup encounters ppERK is expected to be proportional to the amount of ppERK that passes through the nucleus per unit time rather than the concentration of cytoplasmic or nuclear ppERK. Therefore, in our model, the rate of Nup phosphorylation was proportional to the rate of ppERK translocation (See also Supplementary Table 1).

At an early stage after stimulation, ERK is phosphorylated in the cytoplasm. Activated ERK passes across the nuclear envelope with phosphorylation of Nups and interacts with nuclear substrates, resulting in accumulation of ERK in the nucleus. ERK-mediated phosphorylation of Nups enhances ERK import, which works as positive feedback on nuclear import. On the other hand, the nuclear accumulation of ERK does not last long because the available substrates would be decreased by phosphorylation[9], which functions as negative feedback for ERK nuclear

import. Therefore, both positive and negative feedback regulations temporarily emerge. Indeed, positive and negative feedback is a motif observed in excitable systems that is known to inherently generate ultrasensitive responses[10,11]. Therefore, our ERK model could be interpreted as a modified form of the excitable system.

Supplementary References

1. Fujioka, A. *et al.* Dynamics of the Ras/ERK MAPK cascade as monitored by fluorescent probes. *J. Biol. Chem.* **281,** 8917–8926 (2006).

2. Allen, T. D., Cronshaw, J. M., Bagley, S., Kiseleva, E. & Goldberg, M. W. The nuclear pore complex: mediator of translocation between nucleus and cytoplasm. *J. Cell Sci.* **113,** 1651–1659 (2000).

3. Maeshima, K. *et al.* Nuclear pore formation but not nuclear growth is governed by cyclin-dependent kinases (Cdks) during interphase. *Nat. Struct. Mol. Biol.* **17,** 1065–1071 (2010).

4. Aoki, K., Yamada, M., Kunida, K., Yasuda, S. & Matsuda, M. Processive phosphorylation of ERK MAP kinase in mammalian cells. *Proc. Natl. Acad. Sci. U. S. A.* **108,** 12675–12680 (2011).

5. Reiterer, V., Fey, D., Kolch, W., Kholodenko, B. N. & Farhan, H. Pseudophosphatase STYX modulates cell-fate decisions and cell migration by spatiotemporal regulation of ERK1/2. *Proc. Natl. Acad. Sci. U. S. A.* **110,** E2934–2943 (2013).

6. Matsubayashi, Y., Fukuda, M. & Nishida, E. Evidence for existence of a nuclear pore complex-mediated, cytosol-independent pathway of nuclear translocation of ERK MAP kinase in permeabilized cells. *J. Biol. Chem.* **276,** 41755–60 (2001).

7. Whitehurst, A. W. *et al.* ERK2 enters the nucleus by a carrier- independent mechanism. *Proc. Natl. Acad. Sci. U. S. A.* **99,** 7496–7501 (2002).

8. Lidke, D. S. *et al.* ERK nuclear translocation is dimerization-independent but controlled by the rate of phosphorylation. *J. Biol. Chem.* **285,** 3092–3102 (2010).

9. Ahmed, S. *et al.* Data-driven modeling reconciles kinetics of ERK phosphorylation, localization, and activity states. *Mol. Syst. Biol.* **10,** 1–14 (2014).

10. Pomerening, J. R., Sontag, E. D. & Ferrell, J. E. Building a cell cycle oscillator: hysteresis and bistability in the activation of Cdc2. *Nat. Cell Biol.* **5,** 346–351 (2003).

11. Alon, U. Network motifs: theory and experimental approaches. *Nat. Rev. Genet.* **8,** 450–461 (2007).
